# CAN’T STOP THINKING ABOUT OTHERS’ PROBLEMS: COUPLING OF DAILY SUPPORT PROVISION AND COGNITIVE INTERFERENCE

**DOI:** 10.1093/geroni/igae098.1976

**Published:** 2024-12-31

**Authors:** Kelly Cichy, Dakota Witzel, Robert Stawski

**Affiliations:** Kent State University, Kent, Ohio, United States; South Dakota State University, Brookings, South Dakota, United States; University of Essex, Colchester, England, United Kingdom

## Abstract

The perceived availability of social support is cited as protective against the adverse effects of stressors and curtails cognitive interference (e.g., worry and rumination) linked to anxiety and depression. Social support exchanges, however, also involve providing support to others, which has costs to emotional well-being. Although receiving support may disrupt worry and rumination, providing support may elicit thoughts of concern about the situations requiring support provision, leading to increased cognitive interference on days when support is provided and among those who provide frequent support. Using data from the third wave of the National Study of Daily Experiences (NSDE 3) we examined associations among daily social support provision and cognitive interference between persons and within persons across days. Given gender differences in social networks and rumination, we also explore whether gender moderates these associations. Across eight consecutive days, respondents (N=1,236, Mage= 62.6, 57% women) reported on their affect, completed a six-item measure of cognitive interference (e.g., how often think about situations that upset you?), and indicated whether they provided emotional support. Multilevel models controlling for age and gender revealed significant within- and between-person associations, where cognitive interference was higher on days individuals provided emotional support compared to non-support days and for those who provided more support (p<.001), independent of daily negative affect. Gender did not moderate the associations. Findings underscore how listening to others’ concerns confers a mental load not accounted for by negative affect, such that cognitive interference may be one mechanism underlying the costs associated with providing support.

